# Utilizing nutrition-related biomarkers to develop a nutrition-related aging clock for the chinese demographic

**DOI:** 10.3389/fnut.2025.1563220

**Published:** 2025-09-12

**Authors:** Ya-Qing Ma, Ya-Min Dang, Lv-Tao Zeng, Xin Gao, Si-Jia Li, Li-Qun Zhang, Jin Li, Xiao-Yang Zhou, Shan-Shan Ren, Hong-Lei Liu, Ruo-Mei Qi, Jing Pang, Ju Cui, Tie-Mei Zhang, Jian-Ping Cai

**Affiliations:** ^1^Fifth School of Clinical Medicine, Peking University, Beijing, China; ^2^The Key Laboratory of Geriatrics, Beijing Hospital, National Center of Gerontology of National Health Commission, Beijing Institute of Geriatrics, Institute of Geriatric Medicine, Chinese Academy of Medical Sciences, Beijing, China; ^3^Department of Clinical Nutrition, Beijing Hospital, National Center of Gerontology, Institute of Geriatric Medicine, Chinese Academy of Medical Sciences, Beijing, China; ^4^Beijing Advanced Innovation Center for Big Data-based Precision Medicine, Capital Medical University, Beijing, China

**Keywords:** the nutrition-related aging clock, bioelectrical impedance analysis, aging biomarkers, oxidative stress markers, interindividual aging variation

## Abstract

**Introduction:**

This study aims to investigate the relationship between nutrition-related biomarkers, body composition, and oxidative stress indicators in the human aging process, so as to provide new insights for understanding individual aging differences and developing targeted intervention strategies.

**Methods:**

A total of 100 healthy participants aged 26–85 years were enrolled. Plasma concentrations of 9 amino acids and 13 vitamins were quantitatively analyzed, along with urinary oxidative stress markers 8-oxoGuo and 8-oxodGuo. Body composition was assessed using bioelectrical impedance analysis (BIA). A nutrition-based aging clock model was constructed using the Light Gradient Boosting Machine algorithm, with model performance evaluated by mean absolute error (MAE) and coefficient of determination (R^2^).

**Results:**

The younger group showed significantly lower levels of oxidative stress markers compared to the older group. Multiple amino acids and vitamins exhibited age-dependent changes in plasma concentrations. The developed aging clock model demonstrated high predictive accuracy, with an MAE of 2.5877 and R^2^ of 0.8807. Correlation analyses further indicated associations between model-predicted biological age and physiological changes reflected in biochemical and physical examination indicators.

**Discussion:**

This study establishes a significant link between nutrition-related biomarkers, oxidative stress, body composition, and aging. The proposed model serves as a reliable tool for predicting biological age and offers a scientific basis for future research on aging mechanisms and personalized interventions.

## 1 Introduction

In response to the challenges posed by an aging population, researchers have focused on developing aging biomarkers for health identification and assessment (1). In recent years, aging clocks constructed based on various biological markers have emerged, aiming to predict an individual’s biological age and monitor the rate of aging (2). Among the existing aging clocks, those based on DNA methylation (3) and plasma proteome (4) are particularly notable.

Nutrition refers to the process by which the body meets its physiological needs through the intake and metabolism of food. This includes both essential macronutrients (such as proteins, fats, and carbohydrates) and micronutrients (such as vitamins and minerals) (5). Nutrition involves not only caloric intake but also the balance of many biomolecules essential for maintaining physiological function, enhancing health, and preventing diseases (6). Nutritional assessment is a comprehensive process that analyzes nutrition-related health issues by collecting data on food intake and metabolism, alongside biochemical indicators, physical examination results, and other relevant information (7–9). Nutritional science plays a crucial role in promoting healthy aging. Healthy aging not only refers to extending lifespan but also, more importantly, to increasing the number of years of healthy life expectancy (10). The adequacy of nutrition directly influences the health condition and quality of life of the elderly (11). Poor nutritional status in the elderly increases the risk of aging-related chronic diseases. Deficiencies in vitamin B6, B12, and folic acid, for instance, are associated with cognitive decline and Alzheimer’s disease (5, 7, 12). Therefore, nutritional status significantly impacts the aging process, and developing a nutrition-related aging assessment clock is essential for a deeper understanding of aging.

Plasma levels of amino acids and vitamins are closely related to an individual’s nutritional and health status. Tappia et al.’s research indicates that specific vitamins, such as vitamin C and vitamin B6, may help prevent cardiovascular diseases in high-risk individuals (13). Bioelectrical impedance analysis (BIA), a non-invasive technology, can help identify age-related nutritional and metabolic changes, including key indicators such as basal metabolic rate (BMR), muscle mass, total body water, and extracellular water (14, 15). Whether these indicators could contribute to the aging clock establishment remain unknown.

Oxidative stress is closely linked to nutritional status. The free radical theory of aging is one of the most widely known aging theories, with oxidative stress being a key factor in cellular damage and aging (16, 17). Recent research indicates that 8-oxoguanosine (8-oxoGuo) and 8-oxodeoxyguanosine (8-oxo-dGuo) are significant indicators of oxidative stress and aging (18–23). Canfield et al.’s research suggests that reducing oxidative stress may contribute to the positive correlation between free amino acid levels and lifespan (24). Good nutritional status helps neutralize oxidative stress, supports neuroplasticity, and positively impacts recovery outcomes after a stroke. Conversely, malnutrition or poor nutritional status can exacerbate oxidative stress, leading to inflammatory responses and damaging health (25). Therefore, we included the oxidative stress markers 8-oxoGuo and 8-oxo-dGuo in urine as part of the aging clock related to nutritional status.

This study aims to develop an aging clock based on nutrition-related biomarkers, including amino acids and vitamin levels in plasma, body composition, and oxidative stress markers in urine, to assess biological age in individuals. This model has the potential to reveal variations in aging rates between individuals. The construction of this model has significant implications for designing tailored aging intervention strategies and offers new perspectives on the biological basis of aging.

## 2 Materials and methods

### 2.1 Study design and study participants

**FIGURE 1:**
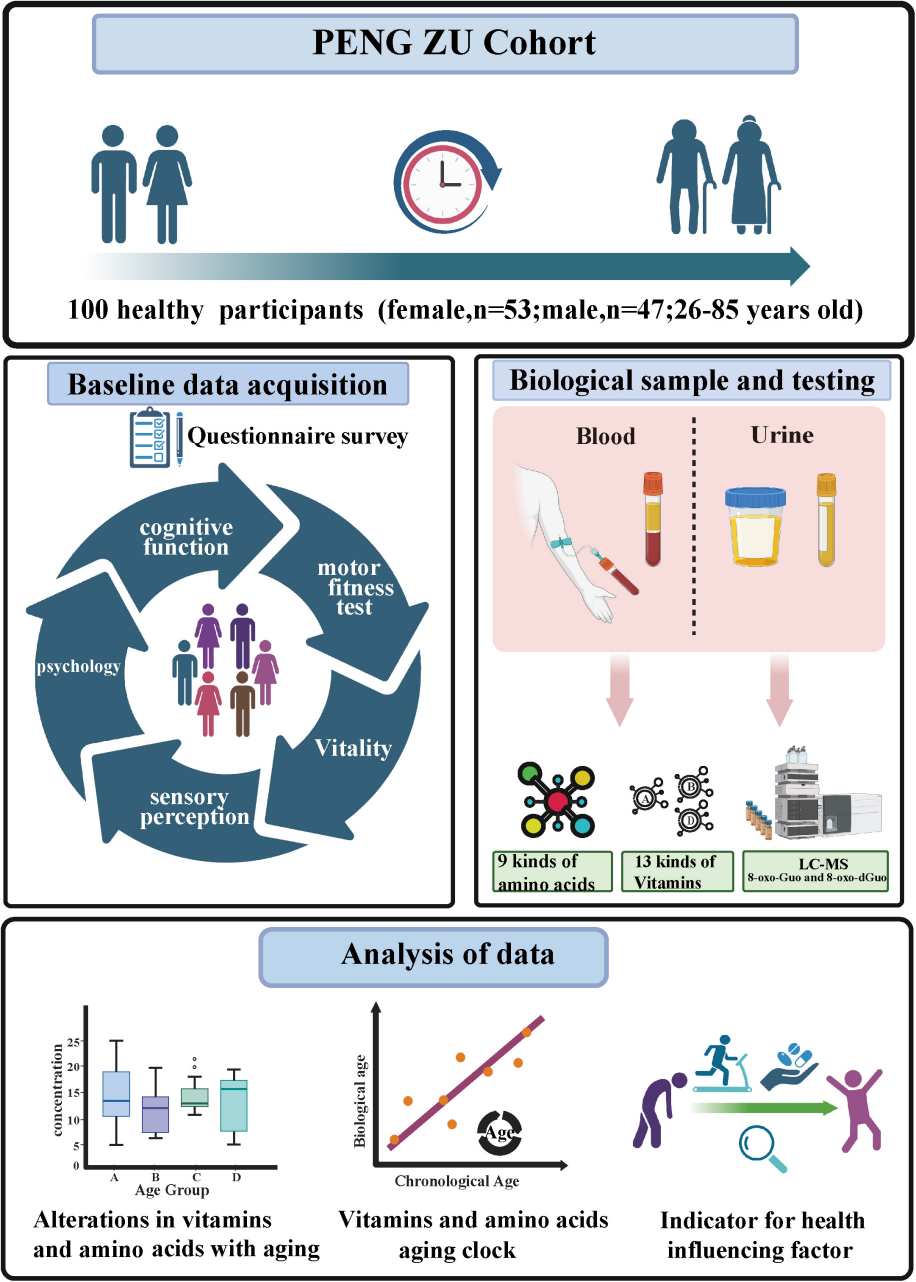
The study outline (Figure created with BioRender.com).

The Ethics Committee of Beijing Hospital approved the protocol for this study (Approval No. 2019BJYYEC-054-02), and the study was conducted in accordance with the Declaration of Helsinki. Each participant provided a signed informed consent form after receiving comprehensive information about the study’s objectives, methods, and potential risks. The PENG ZU cohort is a health aging cohort study covering populations from seven major regions of China (26). We randomly selected 100 healthy volunteers, aged 26–85 years, for this study. The participants were from various age groups. Individuals with serious chronic illnesses or other health issues that could affect the research results were excluded. To ensure that the sample accurately represented a broad range of age demographics and genders, we employed random sampling techniques. The study outline is shown in Figure 1.

### 2.2 Biomarker assessment

#### 2.2.1 Plasma sample analysis

The quantitative analysis of 9 amino acids and 13 vitamins was performed using liquid chromatography-tandem mass spectrometry (LC-MS/MS). The measured amino acids include ethanolamine, L-serine, L-proline, L-cystine, taurine, L-aspartic acid, L-arginine, L-histidine, and 1-methyl-L-histidine. The vitamins include vitamin B1, B2, B3, B5, B6, B7, 5-methyltetrahydrofolate, vitamin A, D2, D3, E, K1, and MK4.

#### 2.2.2 Urine sample analysis

The levels of 8-oxodGuo and 8-oxoGuo in the urine were measured using liquid chromatography-tandem mass spectrometry (LC-MS/MS). The urine creatinine concentration was determined using the Jaffe reaction method with a 7,600 series automatic biochemical analyzer (Hitachi, Japan), following the manufacturer’s instructions. To determine the levels of oxidative stress, we used the 8-oxodGuo/Cre and 8-oxoGuo/Cre ratios. Urine samples were promptly preserved at −80 °C after being collected midstream in the morning. Prior to analysis, the samples were thawed, warmed in a 37 °C water bath for 5 min, centrifuged at 7,500 g for 5 min, and the supernatant was collected (27). To each 200 μL of supernatant, 200 μL of working solution (70% methanol, 30% water, 0.1% formic acid, 5 mmol/L ammonium acetate) was added, along with 10 μL of internal standard 8-oxo-[^15^N_5_]dGuo and 10 μL of internal standard 8-oxo-[^15^N_2_^13^C_1_]Guo (both at a concentration of 240 pg/μL). The mixture was incubated at 37 °C for 10 min and then centrifuged at 12,000 g for 15 min. The samples were separated using an Agilent 1290 UPLC connected to an Agilent 6490 triple quadrupole mass spectrometer (MS/MS) for detection.

#### 2.2.3 Biochemical parameters

In addition to the aforementioned biomarker analyses, we conducted routine biochemical parameter tests on plasma and urine samples, quantitatively analyzing a range of metabolites to comprehensively assess participants’ health status.

### 2.3 BIA

Body composition was assessed using the BCA-2A bioelectrical impedance analyzer (BIA; Tsinghua Tongfang Co., Ltd., Beijing, China). The device operates at frequencies of 5, 50, 100, 250, and 500 kHz, collecting comprehensive bioimpedance data. A set of eight-point contact electrodes was used for six-channel whole-body testing, ensuring measurement accuracy and uniformity. The primary criteria measured included basal metabolic rate, muscle mass, total body water, extracellular water, intracellular water, fat mass, and visceral fat. Participants were instructed to stand barefoot on the electrode plate of the equipment, with their arms abducted at approximately 30 degrees in a standard posture. Intense exercise was prohibited before the assessment. Qualified personnel conducted all measurements following established procedures to ensure consistency and reliability.

### 2.4 Developing an aging clock model using machine learning approaches

This study employed a systematic approach to construct a nutrition-related aging clock model for predicting biological age. The dataset was randomly divided into a training set (70%) and a test set (30%) to evaluate the model’s generalization ability. Five machine learning algorithms were selected for model construction: gradient boosting, LASSO, Light Gradient Boosting Machine (LightGBM), random forest, and XGBoost. All models were implemented using machine learning packages such as caret and XGBoost in R software (version 4.4.1).

To enhance the interpretability and predictive accuracy of the models, we performed feature selection to identify features that significantly contribute to predictions. Additionally, we optimized the models by adjusting parameters such as the number of trees, depth, and learning rate. Using cross- validation and grid search, we determined the optimal parameters to achieve the lowest root mean square error. The optimized models were then used to predict the training set, test set, and entire dataset using the predict function. Model performance was evaluated using the coefficient of determination (R^2^) and mean absolute error (MAE), where R^2^ measures explanatory power and MAE reflects predictive accuracy.

In this study, we defined the age difference (AgeDiff) as the difference between predicted age and actual age. The locally weighted scatterplot smoothing (LOESS) method was applied to regress AgeDiff against age, resulting in the corrected age difference (cAgeDiff): cAgeDiff = AgeDiff–LOESS (AgeDiff∼Age) (28, 29). This method quantifies the predictive bias of the model and categorizes the study subjects into subgroups with different aging rates based on the quartiles of cAgeDiff, providing a new perspective for understanding interindividual differences in aging. Study participants were categorized into subgroups with different aging rates based on the quartiles of cAgeDiff. Those with cAgeDiff values in the bottom quartile (< Q1) were classified as the “decelerated aging” group; those in the middle two quartiles (Q1 ≤ cAgeDiff ≤ Q3) as the “normal aging” group; and those in the top quartile (> Q3) as the “accelerated aging” group.

### 2.5 Statistical analysis

The statistical analysis for this study, including the establishment of the nutrition-related aging clock, was carried out as described in section “2.4 Developing an aging clock model using machine learning approaches.” Data were processed using SPSS 23.0 (SPSS Inc., Chicago, IL, United States) and GraphPad Prism 8 (GraphPad Inc., San Diego, CA, United States). First, the normality of the data was assessed using the Shapiro-Wilk test. If the data did not meet normality, the Kruskal-Wallis H test and Dunn’s *post hoc* comparison were employed. Homogeneity of variance was tested using Levene’s test to satisfy the assumptions of ANOVA; if not met, Welch’s ANOVA was used. After identifying significant differences between groups, multiple comparisons were conducted using the least significant difference or Tamhane’s T2 method. Additionally, the correlation between variables was analyzed using Pearson and Spearman rank correlation coefficients. The level of statistical significance was set at *P* < 0.05.

## 3 Results

### 3.1 Characteristics of the studied cohort

This study included a healthy population across different age groups to construct a nutrition-related aging clock (Table 1 and Supplementary Table 1). We stratified the participants into four age groups based on the median (range): the young group [31 years (26–33)], the young and middle-aged group [45 years (42–48)], the middle-aged group [59 years (56–63)], and the senior group [77.5 years (73–85)]. The sex ratio in each group was relatively equitable, with men constituting between 43.75% and 50.00%. No notable variations in body mass index (BMI) were found across the groups (*P* = 0.551). Differences in drinking and smoking behaviors among age groups were not statistically significant (*P* = 0.588 and *P* = 0.555). However, significant disparities in educational attainment and marital status were observed (*P* < 0.01 and *P* < 0.001), reflecting sociodemographic differences across age cohorts. Psychological stress was more common in the young and middle-aged cohorts (*P* < 0.001), although drinking habits showed no significant variation across the groups (*P* = 0.206). Vegetable eating habits and exercise habits showed a statistically significant difference across age groups (*P* < 0.001). Anthropometric measures and functional assessments revealed physiological changes associated with aging, including significant differences in grip strength (*P* < 0.01) and the light response test (*P* < 0.001). Participants also underwent BIA, which measured key parameters such as total body water, muscle mass, extracellular water, intracellular water, fat mass, and visceral fat, among 35 others (Supplementary Table 2 and Supplementary Figure 1), which provided valuable information for assessing body composition and nutritional status.

**TABLE 1:** Characteristics of study participants.

Baseline characteristics	Young group	Young and middle-aged group	Middle-aged group	Senior group	*P*-value
Number of cases	28	30	26	16	NA
Age (year)	31 (26–33)	45 (42–48)	59 (56–63)	77.50 (73–85)	NA
Sex: male, *n* (%)	13 (46.43%)	15 (50.00%)	12 (46.15%)	7 (43.75%)	NA
Weight	65.85 (42.2–92.3)	70.20 (53.70–104.30)	66.10 (49.60–86.20)	57.85 (46.00–79.10)	0.064
Height	1.686 ± 0.067	1.687 ± 0.077	1.653 ± 0.067	1.595 ± 0.086	< 0.01
BMI (kg/m^2^)	23.32 (16.08–30.49)	23.89 (19.49–32.09)	24.56 (19.81–30.20)	22.67 (17.53–27.58)	0.551
Highest education, *n* (%)					< 0.01
Primary or below	0 (0.00)	0 (0.00)	1 (4.00)	1 (6.25)	NA
Middle school or high school	0 (0.00)	0 (0.00)	5 (20.00)	5 (31.25)	NA
College degree or above	28 (100)	30 (100)	19 (76.00)	10 (62.50)	NA
Marital status, *n* (%)					< 0.001
Spinsterhood	10 (35.71)	1 (3.33)	1 (4.00)	0 (0.0)	NA
Married	18 (64.29)	29 (96.67)	24 (96.00)	16 (100.0)	NA
Psychological stress: yes, *n* (%)	19 (67.86)	21 (70.00)	8 (30.77)	2 (12.50)	< 0.001
Vegetable eating habits, *n* (%)					< 0.001
Occasionally	4 (14.29)	0 (0.0)	3 (11.54)	0 (0.0)	NA
Often	9 (32.14)	15 (50.00)	7 (26.92)	2 (12.50)	NA
Every day	15 (53.57)	15 (50.00)	16 (61.54)	14 (87.50)	NA
Physical exercise habits: yes, *n* (%)	10 (35.71)	18 (62.07)	23 (88.46)	14 (87.50)	< 0.001
Left hand grip strength	29.45 (18.70–57.30)	32.80 (20.70–58.90)	30.83 (21.70–47.60)	23.72 (12.30–39.10)	< 0.01
Right hand grip strength	33.90 (21.60–62.10)	36.45 (23.20–64.20)	30.60 (22.50–50.60)	22.05 (15.70–41.30)	< 0.01
Light reaction test (hand)	0.23 (0.19–0.37)	0.26 (0.20–0.49)	0.29 (0.20–0.82)	0.41 (0.21–1.02)	< 0.001
Light reaction test (foot)	0.30 (0.21–0.44)	0.29 (0.22–0.49)	0.32 (0.21–0.60)	0.47 (0.28–0.95)	< 0.001

Data are expressed as mean ± standard deviation for variables with normal distribution, as the median (minimum–maximum) for variables with non-normal distribution, and as *n* (%) for categorical variables. BMI, body mass index.

### 3.2 Differential analysis of plasma amino acids and vitamins in four age groups, as well as biomarkers in urine samples

**FIGURE 2:**
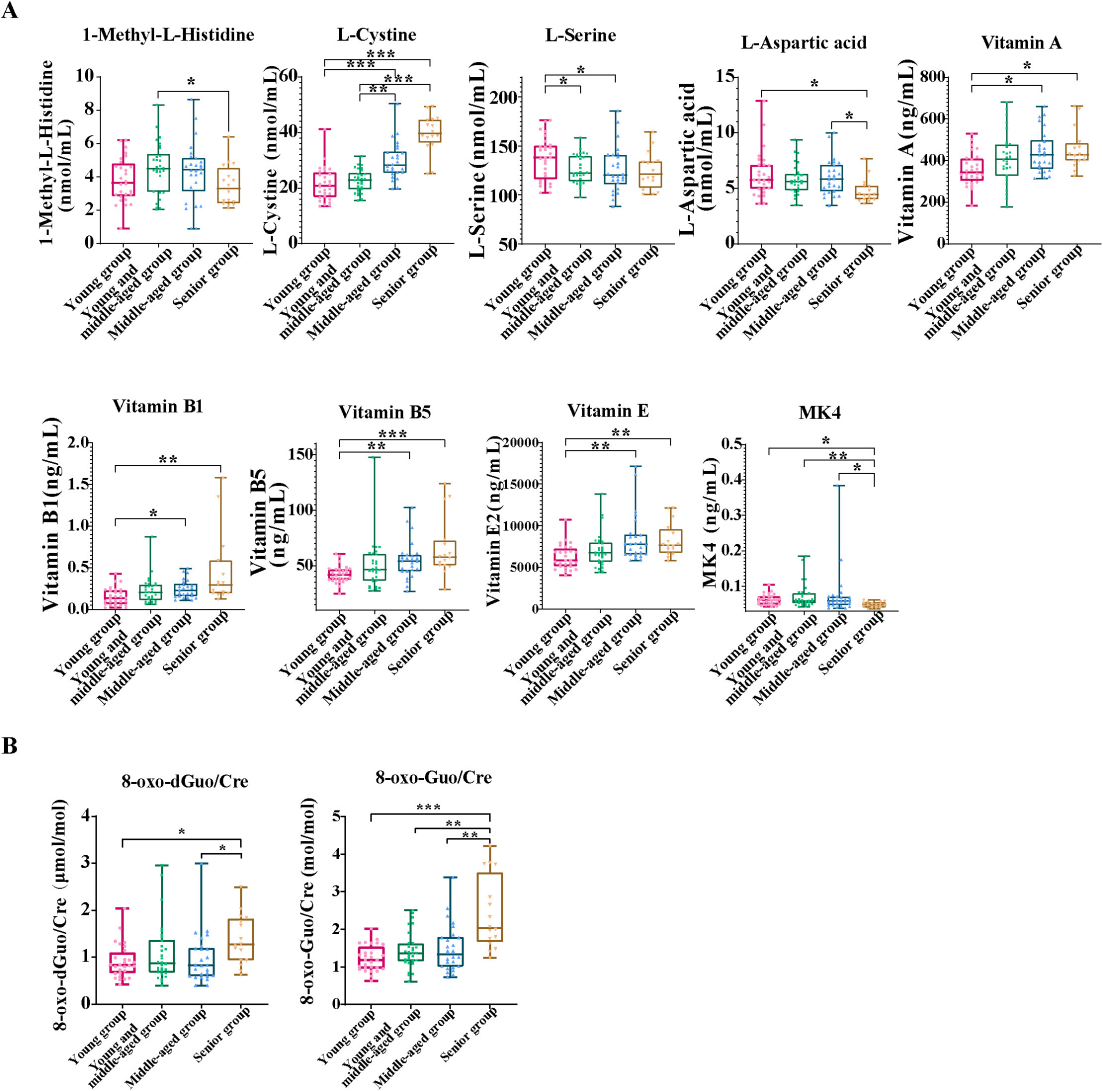
Analysis of biomarker differences in urine and blood samples across the four age groups. **(A)** This panel presents a comparative analysis of amino acid and vitamin biomarkers, with significant differences between age groups. **(B)** This box plot displays the change of urinary oxidative stress markers 8-oxodGuo/Cre and 8-oxoGuo/Cre ratios with age. Data are presented as median (interquartile range). For variables with normal distribution and homogeneity of variance, one-way analysis of variance (ANOVA) was performed, followed by Tukey’s HSD test for *post hoc* comparisons. For variables that did not meet the assumptions of normality or homogeneity of variance, the Kruskal-Wallis test was used, followed by Dunn’s test with Bonferroni correction for *post hoc* comparisons. A significance level of *P* < 0.05 was applied. All statistical analyses were conducted using SPSS version 23.0. Asterisks indicate statistical significance: ***P < 0.001, **P < 0.01, and *P < 0.05.

In this study, we compared the levels of 9 amino acids and 13 vitamins in plasma across four age groups, as well as the levels of oxidative stress markers 8-oxoGuo and 8-oxo-dGuo in the urine, to explore the physiological differences among the groups (Figure 2).

A comparison of amino acid and vitamin markers that exhibit significant differences across age groups is illustrated (Figure 2A). We evaluated the influence of age on the concentrations of these biomarkers. The results indicated that the concentrations of vitamins A, B1, B5, E, and L-cystine progressively increased with age, with the elderly showing the highest concentrations, particularly of L-cystine and vitamin B5. This indicated significant differences between the younger and senior groups (*P* < 0.001). Conversely, the concentrations of 1-methyl-L-histidine, L-aspartic acid, MK4, and L-serine decreased with age, reaching their lowest values in the senior group. In addition, through Kyoto Encyclopedia of Genes and Genomes (KEGG) pathway enrichment analysis, we found several significantly enriched metabolic pathways, such as the pantothenate and CoA biosynthesis pathway, the vitamin B1 (thiamine) metabolism pathway, and the cysteine and methionine metabolism pathway (Supplementary Figure 2B). These results suggest that significant changes in nutrition-related biomarkers are closely associated with age-related physiological changes. The relevant metabolic processes may play a crucial role in the construction of nutrition-related aging clocks, further supporting the significance of energy metabolism and oxidative stress in the aging process. The Kruskal-Wallis test and Dunn’s *post hoc* test results (Figure 2B) showed that the ratios of 8-oxoGuo/Cre and 8-oxodGuo/Cre in the young group were significantly lower than those in the middle-aged and elderly groups, especially the 8-oxoGuo/Cre ratio, which showed a markedly reduced level (*P* < 0.001). In addition, significant differences were observed between the middle-aged and elderly cohorts, indicating that oxidative stress marker concentrations increase significantly with age, underscoring their importance in the aging processes.

### 3.3 Constructing a nutritional aging clock based on machine learning models of blood amino acids and vitamins

**FIGURE 3:**
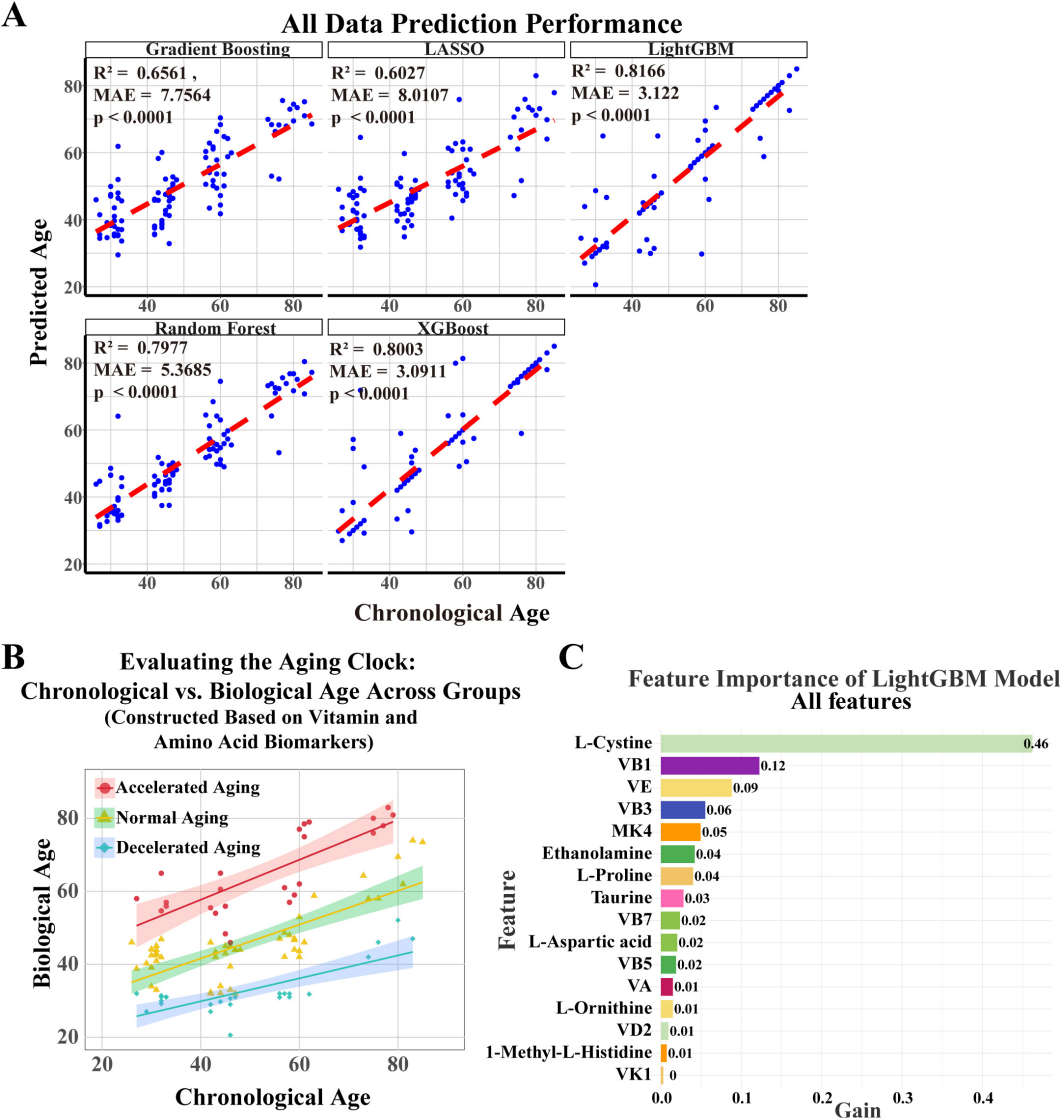
Establishment of an aging clock based on key vitamins and amino acids. **(A)** A nutritional aging clock model was developed using plasma amino acids and vitamins, along with five machine learning algorithms—gradient boosting, LASSO, LightGBM, random forest, and XGBoost—to forecast biological age and evaluate their predictive efficiency. Each subplot shows the algorithm’s predicted results, with blue dots indicating the scatter distribution of projected ages against actual ages, and red lines representing the optimal prediction reference line. **(B)** Based on cAgeDiff, the study participants were divided into three subgroups: accelerated aging, normal aging, and decelerated aging. **(C)** The LightGBM model identified amino acids and vitamins that substantially affect biological age prediction through feature importance analysis.

In this study, we constructed and validated a nutrition-related aging clock model based on amino acids and vitamins detected in plasma, using five machine learning algorithms: gradient boosting, LASSO, LightGBM, random forest, and XGBoost, to predict biological age and evaluate their predictive performance (Figure 3A). Each subplot depicts the predicted results of each individual algorithm, with blue dots representing the scatter distribution of projected ages vs. actual ages and the red line indicating the ideal prediction reference line. The LightGBM model demonstrated exceptional performance, with an R^2^ of 0.8166 and MAE of 3.122 years, demonstrating high predictive accuracy and minimal error.

The LightGBM model proficiently predicts biological age, surpassing other models. Using the corrected age difference (cAgeDiff), we classified the study participants into three subgroups: accelerated aging, normal aging, and decelerated aging (Figure 3B), offering a novel perspective on understanding interindividual variations in aging and potentially facilitating the identification of relevant biomarkers. We then performed a feature significance analysis of the LightGBM model to identify the amino acids and vitamins that most significantly influence the prediction of biological age. The feature significance plot (Figure 3C) showed that L-cystine was the most significant feature, with a gain value of 0.46, emphasizing its crucial role in predicting biological age. The gain values for vitamin B1 and vitamin E were 0.12 and 0.09, respectively, while vitamins B3, MK4, and ethanolamine also had predictive value.

### 3.4 Integration of blood amino acids and vitamins, body composition, and urinary oxidative stress biomarkers: Constructing a multidimensional nutrition-related aging clock

**FIGURE 4:**
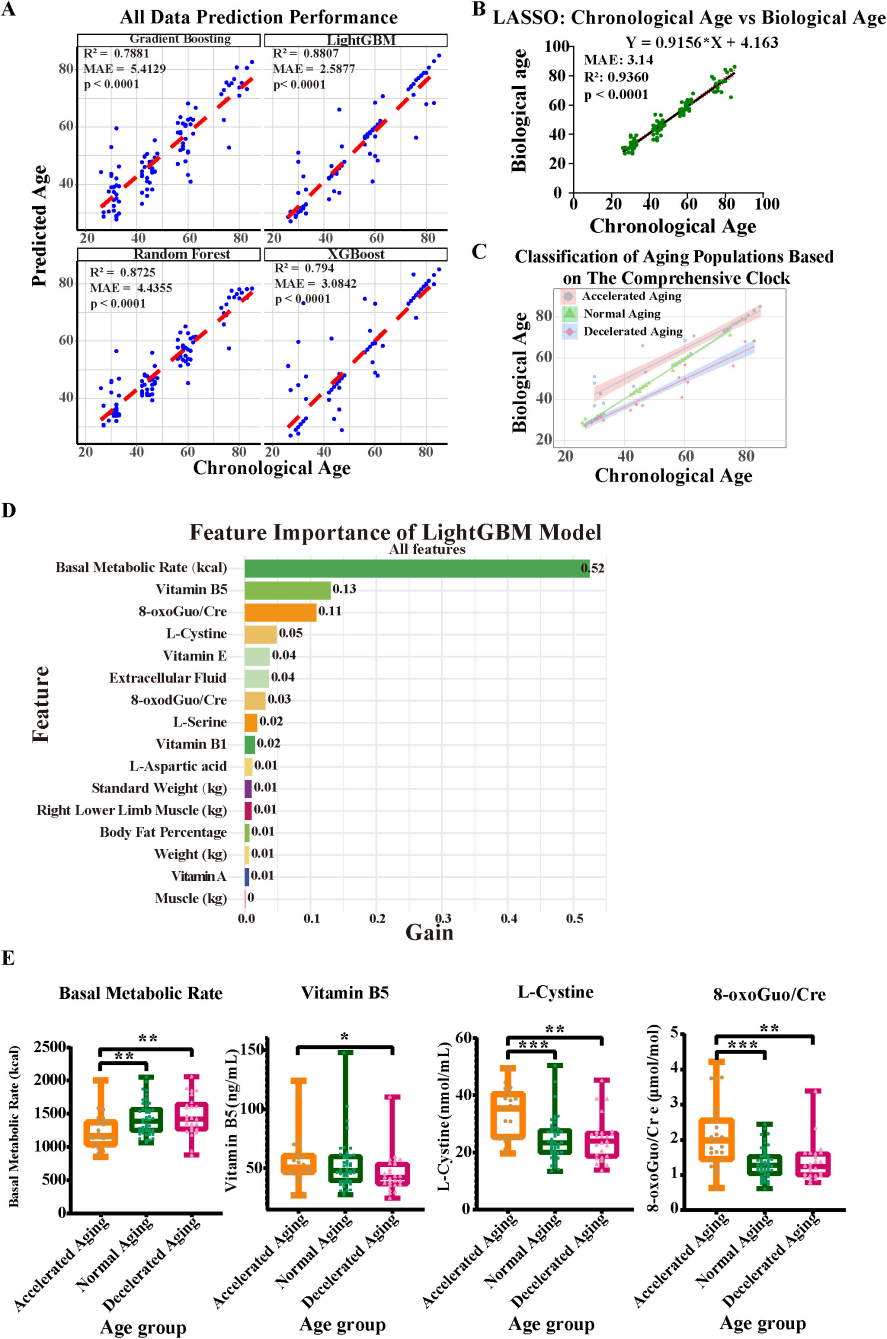
Construction of a comprehensive aging clock based on blood and urine biomarkers and bioelectrical impedance analysis results. **(A,B)** Considering body composition (assessed via BIA), plasma amino acids and vitamins, and urine oxidative stress indicators, five machine learning techniques were employed to develop an aging clock, with the prediction efficacy of each model shown. **(C)** The comprehensive nutritional aging clocks classify populations with different aging rates. **(D)** LightGBM feature-importance ranking (gain) for biological-age prediction. Basal metabolic rate, vitamin B5, 8-oxoGuo/Cre and L-cystine are the top contributors. **(E)** Comparative analysis of key feature factors across different aging groups. Asterisks indicate statistical significance: ***P < 0.001, **P < 0.01, and *P < 0.05.

To improve the predictive accuracy of the model and gain a deeper understanding of the aging process, we comprehensively integrated plasma amino acids and vitamins, body composition (measured by BIA), and urinary oxidative stress markers (Figure 4). The predictive performance of each model is shown (Figures 4A, B). The LightGBM model performed the best, with an R^2^ of 0.8807 and an MAE of 2.5877. The random forest model also performed well, with an R^2^ of 0.8725 and an MAE of 4.3555. The gradient boosting model exhibited average predictive capability, with an R^2^ of 0.7881 and an MAE of 5.4129. The XGBoost model demonstrated significantly lower prediction error than Gradient Boosting (MAE reduced from 5.4129 to 3.0842 years), with a comparable R^2^ of 0.794. Although the LASSO model (Figure 4B) demonstrated high accuracy (R^2^ = 0.9360, MAE = 3.14 years), its feature sparsity may impact generalizability to diverse populations. Consequently, we determined that LightGBM is the most appropriate method for developing the nutritional aging clock.

A demographic categorization based on the comprehensive nutrition-related aging clock, revealing varying rates of aging, is presented (Figure 4C). This classification emphasizes the need to use multiple indicators in aging research and illustrates the diversity of aging among individuals. We then performed a thorough analysis of the importance of each characteristic in the LightGBM model to identify the parameters that most substantially affect the prediction of biological age. The gain values of various features are shown, with the basal metabolic rate at the top having a gain value of 0.52, indicating that it is the most important predictor in the model (Figure 4D). Next were vitamin B5, 8-oxoGuo/Cre, and L-cystine (with gain values of 0.13, 0.11, and 0.05, respectively), which also make significant contributions to the model’s predictive ability. Moreover, features such as vitamin E and extracellular fluid showed certain predictive value, with a gain value of 0.04 each. In light of the substantial contributions of vitamin B5 and L-cystine to the model, yet their unclear biological underpinnings, we performed a systematic functional annotation and pathway-enrichment analysis using the CTD and Metascape databases (see Supplementary Datasheets 1, 2, Supplementary Figures 4A–E and Supplementary Table 3 for full results). These results highlight key biomarkers strongly associated with aging phenotypes, whose predictive importance may reflect underlying biological pathways relevant to age-related decline.

Furthermore, we compared the differences in the model’s important predictive factors—BMR, vitamin B5, 8-oxoGuo/Cre, and L-cystine—among populations with different aging rates (Figure 4E). We found that the BMR in the accelerated aging group was significantly lower than that in the normal and decelerated aging groups (*P* < 0.01). The levels of L-cystine and 8-oxoGuo/Cre were significantly higher in the accelerated aging group than in the normal aging group (*P* < 0.001) and the decelerated aging group (*P* < 0.01). Additionally, the level of vitamin B5 in the accelerated aging group was also higher than that in the decelerated aging group (*P* < 0.05).

### 3.5 Comparative analysis of biochemical indicators in populations with different aging rates

**FIGURE 5:**
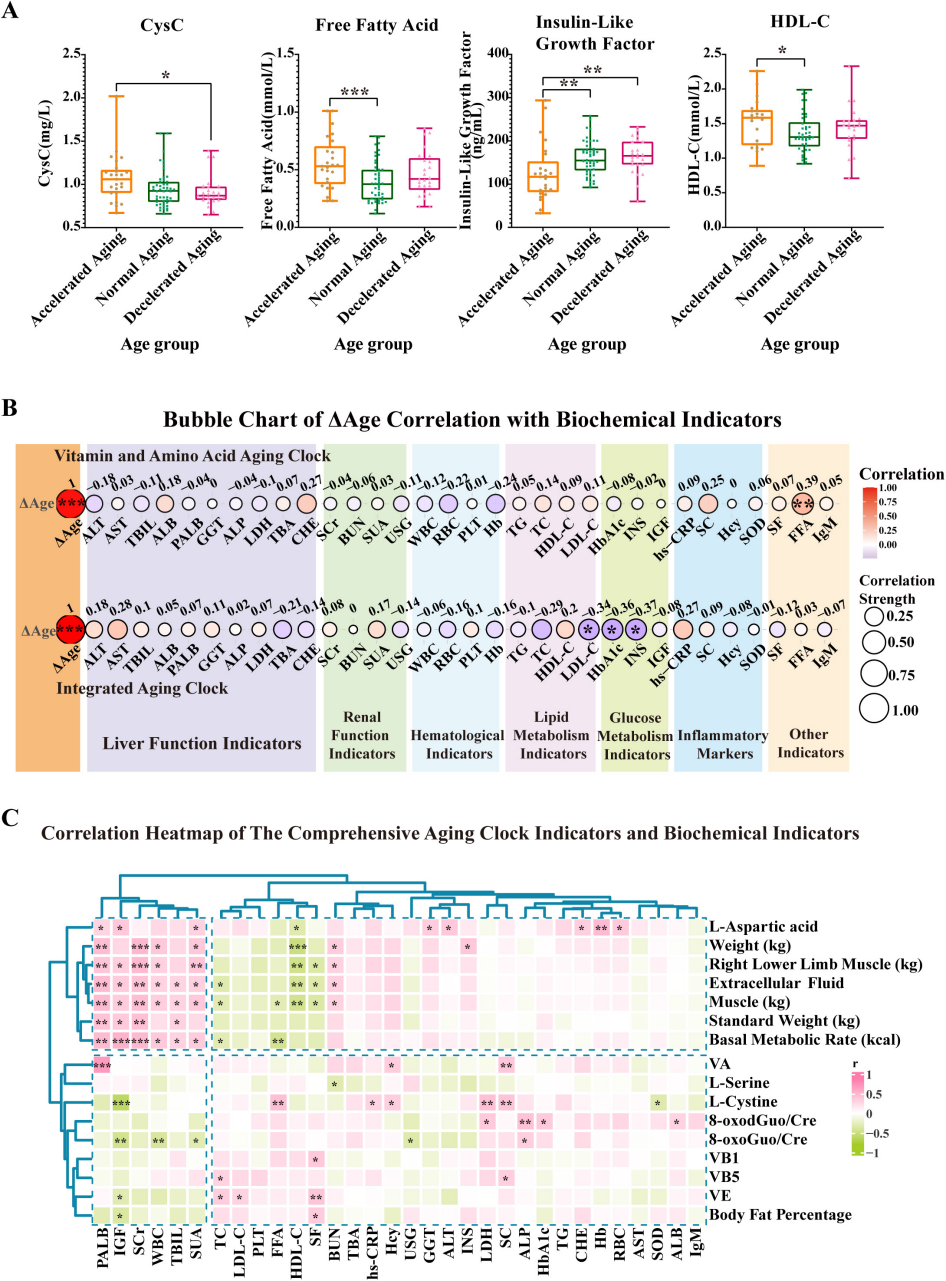
Prediction results of two aging clocks developed from the study population and differential analysis of populations stratified by cAgeDiff (ΔAge). Based on the results from the aging clock grouping, we conducted a comparative analysis of key biochemical indicators in the accelerated, normal, and decelerated aging subgroups. **(A)** This panel presents CysC, FFA, and HDL-C in the accelerated, normal, and decelerated aging groups, respectively. **(B)** This panel illustrates the strength of the association between ΔAge (cAgeDiff) and several biochemical markers. The correlation analysis uses the Spearman correlation coefficient to evaluate the relationship between ΔAge and each biochemical parameter. The two ΔAge values are derived from the vitamin and amino acid aging clock as well as the comprehensive aging clock. **(C)** The heatmap shows how biochemical indicators relate to the full nutritional aging clock markers. The vertical axis represents full nutritional aging clock markers, while the horizontal axis represents biochemical indicators. A color gradient indicates the strength of the correlation coefficient, with green representing a negative correlation and red representing a positive correlation. Asterisks indicate statistical significance: ***P < 0.001, **P < 0.01, and *P < 0.05.

We performed a comparison of important biochemical markers across the three subgroups—accelerated aging, normal aging, and decelerated aging—based on the findings of the nutrition-related aging clock (Figure 5). The levels of free fatty acids (FFA) were significantly higher in the accelerated aging group than in the normal aging group (*P* < 0.001). Similarly, the levels of cystatin C (CysC) were much higher in the accelerated aging group than in the decelerated aging group (*P* < 0.001; Figure 5A). High-density lipoprotein cholesterol (HDL-C) levels were significantly elevated in the accelerated aging group compared with those in the normal aging group (*P* < 0.05). Furthermore, the levels of insulin-like growth factor (IGF) were much lower in the accelerated aging group than in the normal and decelerated aging groups (*P* < 0.01; Figure 5A).

The correlation strength between ΔAge (cAgeDiff) and various biochemical markers is depicted (Figure 5B). The correlation study used the Spearman correlation coefficient to evaluate the relationship between ΔAge and each biochemical parameter. Research on the vitamin and amino acid aging clock revealed a notable association between FFA and ΔAge (r = 0.39, *P* < 0.01), suggesting that FFA serves as a valuable biomarker for determining an individual’s age. Further analysis of the aging clock found that low-density lipoprotein (LDL-C), glycated hemoglobin (HbA1c), and insulin (INS) all had strong relationships with ΔAge (r values of −0.34, −0.36, and −0.37, respectively; *P* < 0.05). LDL-C, HbA1c, and INS may therefore serve as valuable biomarkers for evaluating the aging process. Our findings reveal that FFA, LDL-C, HbA1c, and INS are biochemical markers most closely associated with ΔAge, suggesting their substantial involvement in the aging process.

To further explore the relationship between the comprehensive nutrition-related aging clock and the body’s biochemical indicators, a correlation analysis was performed. The results show the correlation patterns (Figure 5C and Supplementary Figure 5C). The nutrition-related aging clock characteristics, including weight, lower limb muscle mass, extracellular fluid, muscle mass, standard weight, and BMR, exhibited significant positive correlations with biochemical markers such as prealbumin (PALB), IGF, serum creatinine (SCr), white blood cells (WBC), total bilirubin (TBIL), and serum uric acid (SUA), as indicated by the clustering analysis results. Conversely, substantial negative associations were noted between many indicators of the nutrition-related aging clock and markers such as IGF, HDL-C, WBC, total cholesterol (TC), FFA, and serum folate. These results underscore the significance of biochemical markers such as HDL-C, PALB, IGF, and SCr in evaluating an individual’s nutritional status. The aging clock features did not exhibit significant correlations with other indicators, including cholinesterase (CHE) and TC. Overall, these findings highlight the complex interplay between the comprehensive nutrition-related aging clock and the body’s biochemical profile.

### 3.6 Comparative analysis of physical examination indicators among populations with different aging rates

Physical examinations, which are crucial for assessing personal health as well as reflecting the aging process, have been included in our study. These measures objectively assess mobility decline, musculoskeletal integrity, and nutritional status, providing critical physiological context to complement molecular biomarker data.

**FIGURE 6:**
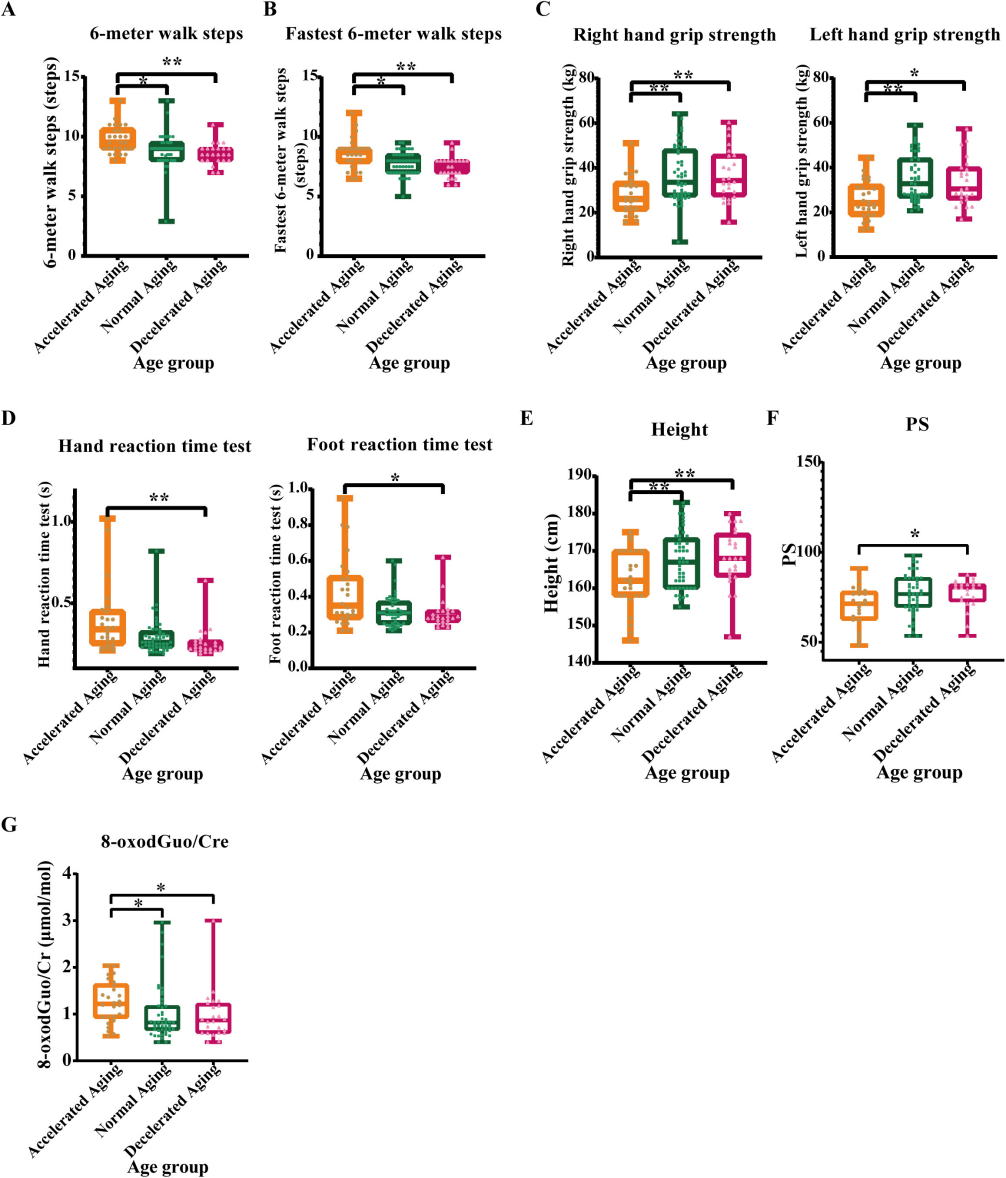
Comparative analysis of physical examination indicators in populations with different aging rates. **(A)** 6-meter walk steps, **(B)** Fastest 6-meter walk steps, **(C)** Grip strength, **(D)** Hand-foot reaction time, **(E)** Height, **(F)** Physiological subhealth score (PS), and **(G)** 8-oxodGuo/Cre (Oxidative stress marker). Physical exams, as a primary method for evaluating individual health status, may reveal physiological changes associated with aging. This study analyzed these physical examination metrics across rapid, normal, and slow aging subgroups. The accelerated aging group performed poorly on multiple physical examination indicators. Asterisks indicate statistical significance: **P < 0.01 and *P < 0.05.

The results of the daily 6 m walk test showed that the number of steps taken by individuals in the accelerated aging group was significantly higher than that taken by those in the normal (*P* < 0.05) and decelerated (*P* < 0.01) aging groups (Figure 6A). The results of the fastest 6 m walking test were similar, with the accelerated aging group showing significantly more steps than the normal and decelerated aging groups (*P* < 0.05 and *P* < 0.01), which indicates that walking ability declines with increasing aging rate (Figure 6B). The grip strength of the accelerated aging group was significantly lower than that of the normal and decelerated aging groups (*P* < 0.01 and *P* < 0.05), indicating a possible correlation with the deterioration of muscle mass and function, as illustrated in Figures 6C. The results of hand and foot reaction time tests showed that the reaction times of the accelerated aging group were significantly longer than those of the other two groups (*P* < 0.01 and *P* < 0.05; Figure 6D), possibly reflecting a decline in nervous system function with increasing aging rate. The height of the accelerated aging group was significantly lower than that of the other two groups (*P* < 0.01), possibly associated with spinal compression or other age-related physical changes (Figure 6E). The physiological subhealth score (PS) (ranging 0–100, higher values indicating better health status) results indicated that the PS score of the accelerated aging group was significantly lower than that of the decelerated aging group (*P* < 0.05; Figure 6F), suggesting a significant association between physiological subhealth status and aging rate. The ratio of 8-oxodGuo/Cre was significantly higher in the accelerated aging group than in the other two groups, indicating that oxidative stress levels increase with the aging rate (Figure 6G).

## 4 Discussion

This study reveals the trends in amino acid and vitamin levels, as well as oxidative stress markers, with age through a comprehensive analysis of plasma and urine samples from participants of different age groups. The results show that the levels of vitamins A, B1, B5, E, and L-cystine significantly increase with age; particularly, differences in L-cystine and vitamin B5 levels between the young and elderly groups are most pronounced, possibly reflecting a decline in metabolic and synthetic functions in the elderly, leading to the accumulation of these molecules in the body. In contrast, the levels of 1-methyl-L-histidine, L-aspartic acid, MK4, and L-serine decrease with age, reflecting a reduction in the activity of specific metabolic pathways (30, 31). These changes in biochemical indicators are closely related to the decline in physiological functions in the elderly population, such as reduced muscle mass and decreased immune function. In urine, 8-oxoGuo/Cre and 8-oxodGuo/Cre levels were significantly lower in the young group than in the middle-aged and senior groups, with significant differences also observed between the middle-aged and senior groups. This indicates that oxidative stress levels significantly increase with age, supporting the viewpoint that oxidative stress plays a key role in the process of aging and its related diseases (18, 19, 32).

In this study, the LightGBM model was the most accurate of the nutrition-related aging clock models created with different machine learning algorithms. Analysis of feature importance revealed that L-cystine, vitamin B1, and vitamin E are significant determinants in the prediction of biological age. These findings highlight the utility of integrating L-cystine, vitamins B1/B5/E, and oxidative stress markers as a novel multimodal panel for aging prediction, advancing our understanding of nutrient-metabolic interplay in aging. By adding BIA, plasma amino acids, vitamins, and urinary oxidative stress markers, the LightGBM model significantly improved its predictive capability (R^2^ = 0.8807, MAE = 2.5877). In this study, BMR, vitamin B5 levels, 8-oxo-Guo/Cre ratio, and L-cystine were identified as key indicators of nutrition-related aging, highlighting the importance of energy metabolism and oxidative stress in aging (21, 33). These indicators may play complex roles in the aging process, with elevated levels potentially serving as biomarkers for the risk of age-related diseases. Changes in their levels not only serve as biomarkers for physiological aging but also associate with adverse health outcomes through pathways requiring further mechanistic investigation. Future research should further explore the specific roles of these biomarkers in the mechanisms of aging and their potential application value in the prevention and treatment of age-related diseases.

Our findings indicate that BMR was significantly lower in the accelerated aging group compared to the normal and decelerated aging groups, suggesting that the rate of decline in BMR may be related to an individual’s aging speed and could serve as a marker to distinguish between different aging rate groups. Furthermore, a research study of senior male populations in southern China showed that an increase in BMR is independently associated with a reduction in all-cause mortality, while BMR decreases non-linearly with age, exhibiting an accelerated decline in older groups (34). BMR decreases with age and is closely related to the aging process. Kitazoe et al. found that mass-specific BMR (msBMR) and renormalized BMR (RmsBMR) can serve as new biomarkers for assessing aging, reflecting metabolic changes during the aging process (35). In summary, these results highlight the importance of basal metabolic rate (BMR) in the aging process. Changes in BMR not only reflect an individual’s aging speed but may also provide clues for identifying different aging types. This finding offers new directions for understanding aging mechanisms and improving health management for the elderly.

Furthermore, our study found that vitamin B5 levels were higher in the accelerated aging group than in the decelerated aging group. Vitamin B5, also known as pantothenic acid, is an essential component for the synthesis of coenzyme A (CoA) and acyl carrier protein. CoA is not only a necessary cofactor for the synthesis of key biomolecules such as fatty acids, cholesterol, acetylcholine, and bile acids but also plays a central role in many metabolic pathways (36, 37). Since humans and animals cannot synthesize pantothenic acid, they must depend on food sources to meet their vitamin requirements. This external reliance emphasizes the importance of pantothenic acid in sustaining health and avoiding associated nutritional deficits (38). Additional studies have revealed that plasma vitamin B5 levels are associated with an increased risk of all-cause mortality, especially among hypertensive patients in China, with this connection being more prominent in the elderly and those with adequate folate levels (39). Regarding whether excessive intake of vitamin B5 can accelerate aging, current research findings are inconsistent. On the one hand, pantothenic acid, as a precursor to coenzyme A, is essential for cellular energy metabolism and antioxidant defense. In theory, having an appropriate quantity of pantothenic acid may help slow the aging process. On the other hand, excessive pantothenic acid consumption may disturb metabolic equilibrium inside cells, resulting in increased oxidative stress and potentially accelerating cellular aging. When examining the link between pantothenic acid consumption and aging, it is vital to compare the potential advantages in terms of health promotion and illness prevention with the potential adverse effects. Future studies should investigate the association between pantothenic acid consumption, metabolic state, and aging, as well as identify strategies to maximize pantothenic acid intake through dietary or supplemental approaches to promote healthy aging.

We also found that the levels of L-cystine were significantly higher in the accelerated aging group than in the normal aging group. L-cystine is a specific amino acid formed by the linkage of two cysteine molecules through a disulfide bond. Lawrence C. Johnson and colleagues investigated the relationship between L-cystine and healthspan indicators related to aging using plasma metabolomics analysis, and discovered that the concentration of L-cystine in the elderly group was significantly higher than that in the young group, which is consistent with the findings of this study (40). Bramer et al. found that in the plasma of patients with mild and severe COVID-19 infections, the levels of L-cystine were significantly elevated (41). Wang et al. by measuring the plasma levels of L-cystine in patients with Attention Deficit Hyperactivity Disorder (ADHD) and healthy control groups, found that the plasma levels of L-cystine in the ADHD group were significantly higher compared to the healthy control group (42). These studies collectively suggest that L-cystine may be related to the severity of the disease, and the increase in L-cystine may reflect the body’s metabolic adaptation in response to oxidative stress and inflammatory responses. In accelerated aging individuals, the increase in L-cystine may be related to the decline in cellular antioxidant capacity and chronic inflammatory states, both of which are closely associated with the increased risk of age-related diseases. Future research should further explore the specific mechanisms of action of L-cystine under different physiological and pathological conditions, as well as its potential applications in aging and disease management.

We found that the ratio of the oxidative stress marker 8-oxoGuo/Cre was abnormally increased in the accelerated aging group. 8-oxo-Guo, indicative of RNA oxidative damage, correlates with elevated oxidative stress levels in several age-related disorders and serves as a crucial biomarker for evaluating oxidative stress status and disease risk (21). Moreover, our findings are consistent with Vatner et al.’s view that oxidative stress is a important mechanism limiting longevity and healthy aging (43).

Additionally, our study demonstrated a markedly increased amount of CysC in the accelerated aging group compared with that in the normal aging group, suggesting that CysC serves as a biomarker for declining renal function associated with accelerated aging. Recent investigations have demonstrated that heightened levels of CysC in individuals with metabolic syndrome correlate with a greater risk of all-cause mortality, including cardiovascular and cancer-related fatalities (44). This highlights the importance of CysC in assessing age-related physiological changes. The accelerated aging group showed markedly increased levels of FFA compared with both the normal and decelerated aging groups. This rise may indicate a disturbance in lipid metabolism, often seen in the elderly, with possible ramifications for the pathophysiology of age-related illnesses, such as type 2 diabetes and cardiovascular disorders (45). The concentration of IGF was much lower in the accelerated aging group than in the normal aging group, which may signify a disruption in growth signaling pathways crucial for maintaining tissue homeostasis and regeneration in older individuals (46, 47). Furthermore, HDL-C levels were markedly elevated in the accelerated aging group compared with those in the normal aging group, suggesting a correlation between HDL-C and accelerated aging. This discovery contradicts previous research, which indicated that elevated levels of HDL-C correlate with lifespan (48). Nevertheless, several studies have revealed that the inverse relationship between HDL-C and ASCVD stabilizes when HDL-C levels approach 40 mg/dL, and excessively elevated HDL-C levels may correlate with heightened risk, demonstrating a U-shaped curve (49, 50). Another study pointed out that higher levels of HDL-C are associated with an increased risk of fractures in healthy elderly individuals (51). Additionally, the SWAN HDL study found that women with midlife HDL-C > 80 mg/dL had 2.3-fold higher risk of cognitive decline over 20 years (52). Our data extend these findings to accelerated aging, where dysfunctional HDL likely promotes multisystem decline (e.g., bone loss, neuroinflammation). Future work should prioritize HDL functional assays over concentration alone.

Ultimately, by analyzing the physical examination metrics of various aging rate subgroups, we elucidated the relationship between aging rate and alterations in physiological function. The findings of the 6-m walking test demonstrated that walking ability diminishes with an increase in the rate of aging. The grip strength test findings indicated that the accelerated aging group had considerably reduced grip strength compared with both the normal and decelerated aging groups, indicating a reduction in muscle mass and function (53). Height measurements indicated that the accelerated aging group had a markedly reduced height compared with the other two groups, may reflect age-related physiological alterations such as spinal compression, rarefaction of bone (54, 55). The results of the response time test further confirmed that brain function declines with advancing age. PS scores were significantly increased in the accelerated aging cohort, and the ratio of oxidative stress markers 8-oxodGuo/Cre to 8-oxoGuo/Cre was disproportionately increased, indicating a decline in the individual’s physiological subhealth status and elevated oxidative stress levels during the aging process (43, 56). The accelerated aging group had worse performance in many physical examination parameters, possibly associated with increased oxidative stress and reduced physiological function.

Physical examination indicators and biochemical markers are important tools for determining individual aging rates, and their patterns may suggest prospective targets for anti-aging therapies. Specific exercise programs may be created to enhance muscle strength and improve physical function in response to decreasing grip strength and walking ability; in response to increased oxidative stress, antioxidant treatment may be explored to slow the aging process (57, 58). Future research should delve deeper into the associations between these biomarkers and aging rates, evaluating corresponding intervention measures with the aim of developing more effective prevention and treatment strategies.

This study has limitations including its modest sample size (*n* = 100), limited elderly subgroup (*n* = 16), and restricted generalizability to chronic disease populations due to strict health screening. Findings are expressly applicable to demographically similar, strictly defined healthy populations and cannot be directly extrapolated to individuals with chronic diseases. while our feature selection approach identified biomarkers highly predictive of biological age, the cross-sectional design precludes causal inference. Despite this, our findings provide valuable baseline data on nutrition-related aging biomarkers in healthy adults. Future research should first validate these findings in larger cohorts with adequate elderly representation and chronic disease populations, before advancing to: mechanistic exploration of these biomarkers, personalized interventions, aging risk modeling, and novel anti-aging therapies. And longitudinal studies should validate whether these biomarkers modulate aging trajectories or primarily reflect age-related physiological changes. This progression will enable targeted strategies to improve healthy aging outcomes.

## Data Availability

The raw data supporting the conclusions of this article will be made available by the authors, without undue reservation.
