# Supplementary material for: Utilizing nutrition-related biomarkers to develop a nutrition-related aging clock for the chinese demographic
Source: Front Nutr. 2025 Sep 12;12:1563220. doi: 10.3389/fnut.2025.1563220 (PMC12465629; doi:10.3389/fnut.2025.1563220)
Supplement: Supplementary file 1 [file Supplementary_file_1.docx]

Supplementary Material

# Supplementary Figures and Tables

## Supplementary Tables

**Supplementary Table S1.** Baseline characteristics of the study population.

| Baseline characteristics | Young group | Young and middle-aged group | Middle-aged group | Senior group | *P* -value |
| --- | --- | --- | --- | --- | --- |
| Number of cases | 28 | 30 | 26 | 16 | NA |
| Age (year) | 31 (26–33) | 45 (42–48) | 59 (56–63) | 77.50 (73–85) | NA |
| Sex: male, n (%) | 13 (46.43%) | 15 (50.00%) | 12 (46.15%) | 7 (43.75%) | NA |
| Weight | 65.85 (42.2–92.3) | 70.20 (53.70–104.30) | 66.10 (49.60–86.20) | 57.85 (46.00–79.10) | 0.064 |
| Height | 1.686±0.067 | 1.687±0.077 | 1.653±0.067 | 1.595±0.086 | <0.01 |
| BMI (kg/m^2^) | 23.32 (16.08–30.49) | 23.89 (19.49–32.09) | 24.56 (19.81–30.20) | 22.67 (17.53–27.58) | 0.551 |
| Drinking alcohol status, n (%) |  |  |  |  | 0.588 |
| Never | 22 (78.57) | 23 (76.67) | 22 (84.62) | 16 (100.00) | NA |
|  |  |  |  |  |  |
| Now drinks regularly | 4 (14.29) | 5 (16.67) | 3 (11.54) | 0 (0.00) | NA |
|  |  |  |  |  |  |
| used to drink, but quit now | 2 (7.14) | 2 (6.67) | 1 (3.85) | 0 (0.00) | NA |
|  |  |  |  |  |  |
| Smoking status, n (%) |  |  |  |  | 0.555 |
| Never smoked | 23 (82.14) | 23 (76.67) | 21 (80.77) | 14 (87.50) | NA |
|  |  |  |  |  |  |
| Current smokers | 2 (7.14) | 5 (16.67) | 4 (15.38) | 0 (0.00) | NA |
|  |  |  |  |  |  |
| Former smokers and current quitters | 3 (10.71) | 2 (6.67) | 1 (3.85) | 2 (12.50) | NA |
|  |  |  |  |  |  |
| Highest education, n (%) |  |  |  |  | <0.01 |
| Primary or below | 0 (0.00) | 0 (0.00) | 1 (4.00) | 1 (6.25) | NA |
| Middle school or high school | 0 (0.00) | 0 (0.00) | 5 (20.00) | 5 (31.25) | NA |
| College degree or above | 28 (100) | 30 (100) | 19 (76.00) | 10 (62.50) | NA |
| Marital status, n (%) |  |  |  |  | <0.001 |
| Spinsterhood | 10 (35.71) | 1 (3.33) | 1 (4.00) | 0 (0.0) | NA |
| Married | 18 (64.29) | 29 (96.67) | 24 (96.00) | 16 (100.0) | NA |
| Psychological stress: Yes, n (%) | 19 (67.86) | 21 (70.00) | 8 (30.77) | 2 (12.50) | <0.001 |
| Water drinking habit, n (%) |  |  |  |  | 0.206 |
| Water | 21 (75.00) | 13 (43.33) | 14 (53.85) | 11 (68.75) | NA |
| Coffee | 2 (7.14) | 5 (16.67) | 3 (11.54) | 0 (0.00) | NA |
| Beverages | 1 (3.57) | 0 (0.00) | 0 (0.0) | 0 (0.00) | NA |
| Tea | 4 (14.29) | 12 (40.00) | 9 (34.62) | 5 (31.25) | NA |
|  |  |  |  |  |  |
| Eat fruit habit, n (%) |  |  |  |  | 0.063 |
| Never | 0 (0.00) | 0 (0.00) | 0 (0.00) | 1 (6.25) | NA |
| Occasionally | 10 (35.71) | 4 (13.33) | 2 (7.69) | 1 (6.25) | NA |
| Often | 7 (25.00) | 12 (40.00) | 8 (30.77) | 6 (37.50) | NA |
| every day | 11 (39.29) | 14 (46.67) | 16 (53.33) | 8 (50.00) | NA |
| Vegetable eating habits, n (%) |  |  |  |  | <0.001 |
| Occasionally | 4 (14.29) | 0 (0.0) | 3 (11.54) | 0 (0.0) | NA |
| Often | 9 (32.14) | 15 (50.00) | 7 (26.92) | 2 (12.50) | NA |
| Every day | 15 (53.57) | 15 (50.00) | 16 (61.54) | 14 (87.50) | NA |
| Physical exercise habits: Yes, n (%) | 10 (35.71) | 18 (62.07) | 23 (88.46) | 14 (87.50) | <0.001 |
| Time to do housework everyday (hours) | 2.00 (0–8.00) | 1.25 (0.50–16.00) | 1 (0.50–15.00) | 2.5 (1.00–12.00) | 0.087 |
| Total sleep time hour (hours) | 7.00 (6.00–8.00) | 7.00 (5.00–8.00) | 6.50 (4.00–8.00) | 6.00 (4.00–8.00) | 0.074 |
| Waistline | 76.61±9.79 | 81.53±8.75 | 85.08±10.65 | 85.37±9.57 | 0.12 |
| Hipline | 95.79±8.43 | 96.33±5.98 | 97.60±6.35 | 98.44±5.29 | 0.563 |
| Left hand grip strength | 29.45 (18.70–57.30) | 32.80 (20.70–58.90) | 30.83 (21.70–47.60) | 23.72 (12.30–39.10) | <0.01 |
|  |  |  |  |  |  |
| Right hand grip strength | 33.90 (21.60–62.10) | 36.45 (23.20–64.20) | 30.60 (22.50–50.60) | 22.05 (15.70–41.30) | <0.01 |
| Time required for a daily walking speed of 6 m (s) | 4.75 (4.00–7.00) | 4.85 (3.00–8.60) | 4.30 (1.10–6.10) | 5.05 (3.60–8.10) | 0.118 |
| Fastest speed walking 6 m (s) | 3.35 (2.70–5.80) | 3.45 (2.00–6.40) | 3.60 (2.60–5.60) | 3.75 (2.80–4.80) | 0.144 |
| Light reaction test (hand) | 0.23 (0.19–0.37) | 0.26 (0.20–0.49) | 0.29 (0.20–0.82) | 0.41 (0.21–1.02) | <0.001 |
| Light reaction test (foot) | 0.30 (0.21–0.44) | 0.29 (0.22–0.49) | 0.32 (0.21–0.60) | 0.47 (0.28–0.95) | <0.001 |

**Note:** Data are expressed as mean ± standard deviation for variables with normal distribution, as the median (minimum-maximum) for variables with non-normal distribution, and as n (%) for categorical variables.

Abbreviations: BMI: body mass index.

**Supplementary Table S2.** Indicators of bioelectrical impedance analysis (BIA).

| number | Specific Measurement Items of Bioelectrical Impedance Analysis |
| --- | --- |
| 1 | ID |
| 2 | Age |
| 3 | Height (cm) |
| 4 | Weight (kg) |
| 5 | Intracellular fluid |
| 6 | Extracellular fluid |
| 7 | Muscle (kg) |
| 8 | Protein (kg) |
| 9 | Lean body mass (kg) |
| 10 | Bone mass (kg) |
| 11 | Fat (kg) |
| 12 | Fat mass index (FMI) |
| 13 | Body fat percentage |
| 14 | Edema coefficient |
| 15 | Waist-to-hip ratio |
| 16 | Body mass index |
| 17 | Muscle control amount (kg) |
| 18 | Fat control amount (kg) |
| 19 | Weight control amount (kg) |
| 20 | Standard weight (kg) |
| 21 | Basal metabolic rate (kcal) |
| 22 | Trunk muscle (kg) |
| 23 | Trunk bone mass (kg) |
| 24 | Trunk fat (kg) |
| 25 | Left upper limb muscle (kg) |
| 26 | Skeletal muscle mass index (SMMI) |
| 27 | Left upper limb bone mass (kg) |
| 28 | Left upper limb fat (kg) |
| 29 | Right upper limb muscle (kg) |
| 30 | Right upper limb bone mass (kg) |
| 31 | Right upper limb fat (kg) |
| 32 | Left lower limb muscle (kg) |
| 33 | Left lower limb bone mass (kg) |
| 34 | Left lower limb fat (kg) |
| 35 | Right lower limb muscle (kg) |
| 36 | Right lower limb bone mass (kg) |
| 37 | Right lower limb fat (kg) |

**Note:** The study population underwent bioelectrical impedance analysis testing, measuring key parameters including basal metabolic rate, muscle mass, total body water, extracellular water, intracellular water, fat mass, and visceral fat, among 35 indicators, providing important data support for assessing body composition and nutritional status.

Supplementary Table S3. Functional annotation of vitamin B5-interacting gene modules identified by GO-MCODE analysis.

| **Network** | **Annotation** |
| --- | --- |
| hits_SUB1_MCODE_1 | R-HSA-8934593\|Regulation of RUNX1 Expression and Activity\|-9.6;R-HSA-9839394\|TGFBR3 expression\|-8.9;R-HSA-2559585\|Oncogene Induced Senescence\|-8.3 |
| hits_SUB1_MCODE_2 | R-HSA-9662361\|Sensory processing of sound by outer hair cells of the cochlea\|-10.0;R-HSA-9662360\|Sensory processing of sound by inner hair cells of the cochlea\|-9.5;R-HSA-9659379\|Sensory processing of sound\|-9.3 |
| hits_SUB1_MCODE_3 | R-HSA-216083\|Integrin cell surface interactions\|-31.6;M18\|PID INTEGRIN1 PATHWAY\|-29.7;hsa04512\|ECM-receptor interaction\|-28.1 |
| hits_SUB1_MCODE_4 | R-HSA-8856828\|Clathrin-mediated endocytosis\|-18.6; R-HSA-199991\|Membrane Trafficking\|-13.4; R-HSA-8856825\|Cargo recognition for clathrin-mediated endocytosis\|-13.3 |
| hits_SUB1_MCODE_5 | GO:1903034\|regulation of response to wounding\|-5.2; R-HSA-9013149\|RAC1 GTPase cycle\|-5.1; GO:0070372\|regulation of ERK1 and ERK2 cascade\|-4.5 |
| hits_SUB1_MCODE_6 | R-HSA-9013409\|RHOJ GTPase cycle\|-16.6; R-HSA-9012999\|RHO GTPase cycle\|-11.0; R-HSA-9013148\|CDC42 GTPase cycle\|-10.7 |
| hits_SUB1_MCODE_7 | R-HSA-5602498\|MyD88 deficiency (TLR2/4) \|-8.5; R-HSA-5603041\|IRAK4 deficiency (TLR2/4) \|-8.5; R-HSA-5686938\|Regulation of TLR by endogenous ligand\|-8.2 |
| hits_SUB1_MCODE_8 | R-HSA-418594\|G alpha (i) signalling events\|-9.9; R-HSA-388396\|GPCR downstream signalling\|-8.4; R-HSA-372790\|Signaling by GPCR\|-8.2 |
| hits_SUB1_MCODE_9 | M5884\|NABA CORE MATRISOME\|-10.2; R-HSA-2022090\|Assembly of collagen fibrils and other multimeric structures\|-10.2; R-HSA-1474244\|Extracellular matrix organization\|-9.9 |
| hits_SUB1_MCODE_10 | R-HSA-416476\|G alpha (q) signalling events\|-8.6; R-HSA-373076\|Class A/1 (Rhodopsin-like receptors) \|-7.8; hsa04080\|Neuroactive ligand-receptor interaction\|-7.7 |
| hits_SUB1_MCODE_11 | GO:0045332\|phospholipid translocation\|-7.6; R-HSA-936837\|Ion transport by P-type ATPases\|-7.6; GO:0034204\|lipid translocation\|-7.5 |
| hits_SUB1_MCODE_12 | R-HSA-70326\|Glucose metabolism\|-7.1; R-HSA-71387\|Metabolism of carbohydrates and carbohydrate derivatives\|-5.4 |
| hits_SUB1_MCODE_13 | R-HSA-977225\|Amyloid fiber formation\|-7.3; GO:0007162\|negative regulation of cell adhesion\|-5.9 |
| hits_SUB1_MCODE_14 | R-HSA-388844\|Receptor-type tyrosine-protein phosphatases\|-9.6; R-HSA-6794362\|Protein-protein interactions at synapses\|-7.7; GO:0050808\|synapse organization\|-5.7 |
| hits_SUB1_MCODE_15 | WP4222\|Phosphodiesterases in neuronal function\|-8.2; hsa04924\|Renin secretion\|-7.9; hsa00230\|Purine metabolism\|-7.1 |

Note: Functional modules enriched among vitamin B5-interacting genes were extracted with GO-MCODE. For each module, the highest-scoring pathway or GO term is reported together with its −log10(P-value) derived from a hypergeometric test against the whole-genome background. Database prefixes: R-HSA (Reactome), GO (Gene Ontology), WP (WikiPathways), hsa (KEGG).

## Supplementary Figures


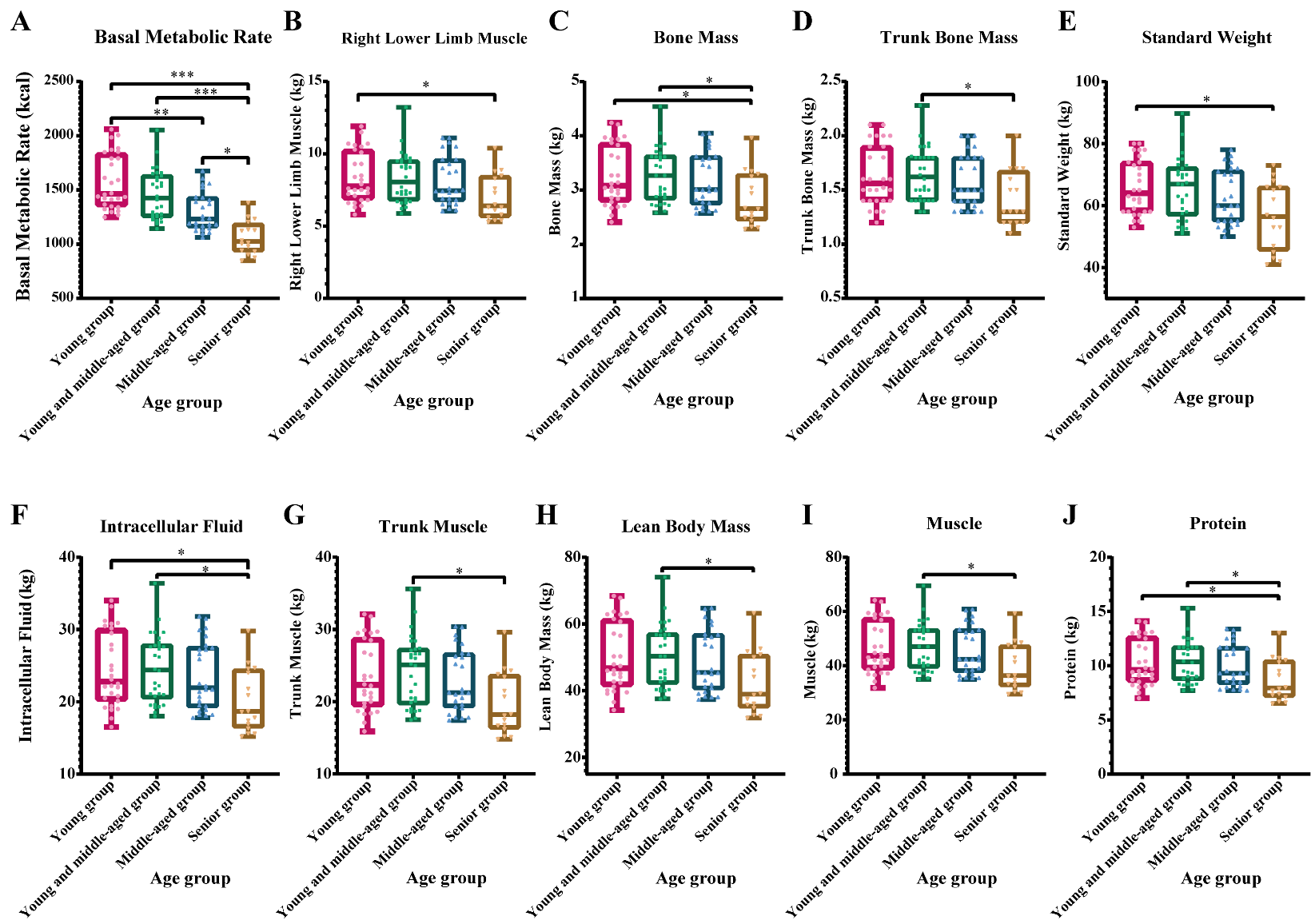


**Supplementary Figure 1.** Bioelectrical impedance analysis indicators with significant differences among the four age groups. This figure comprehensively examines the age-related differences in A) basal metabolic rate, B) right lower limb muscle, C) bone mass, D) trunk bone mass, E) standard weight, F) intracellular fluid, G) trunk muscle, H) lean body mass, I) muscle, and J) protein across various age groups. This figure shows that basal metabolic rate declines significantly with age, with the young group having the highest rate and the senior group exhibiting the lowest, reflecting the profound impact of aging on the body's resting energy requirements. These parameters cover a wide range of metabolic, body composition, and fluid balance aspects, comprehensively reflecting the physiological changes that occur with aging. This figure clearly demonstrates the differences in these indicators among the various age groups, providing important insights into the human aging process.


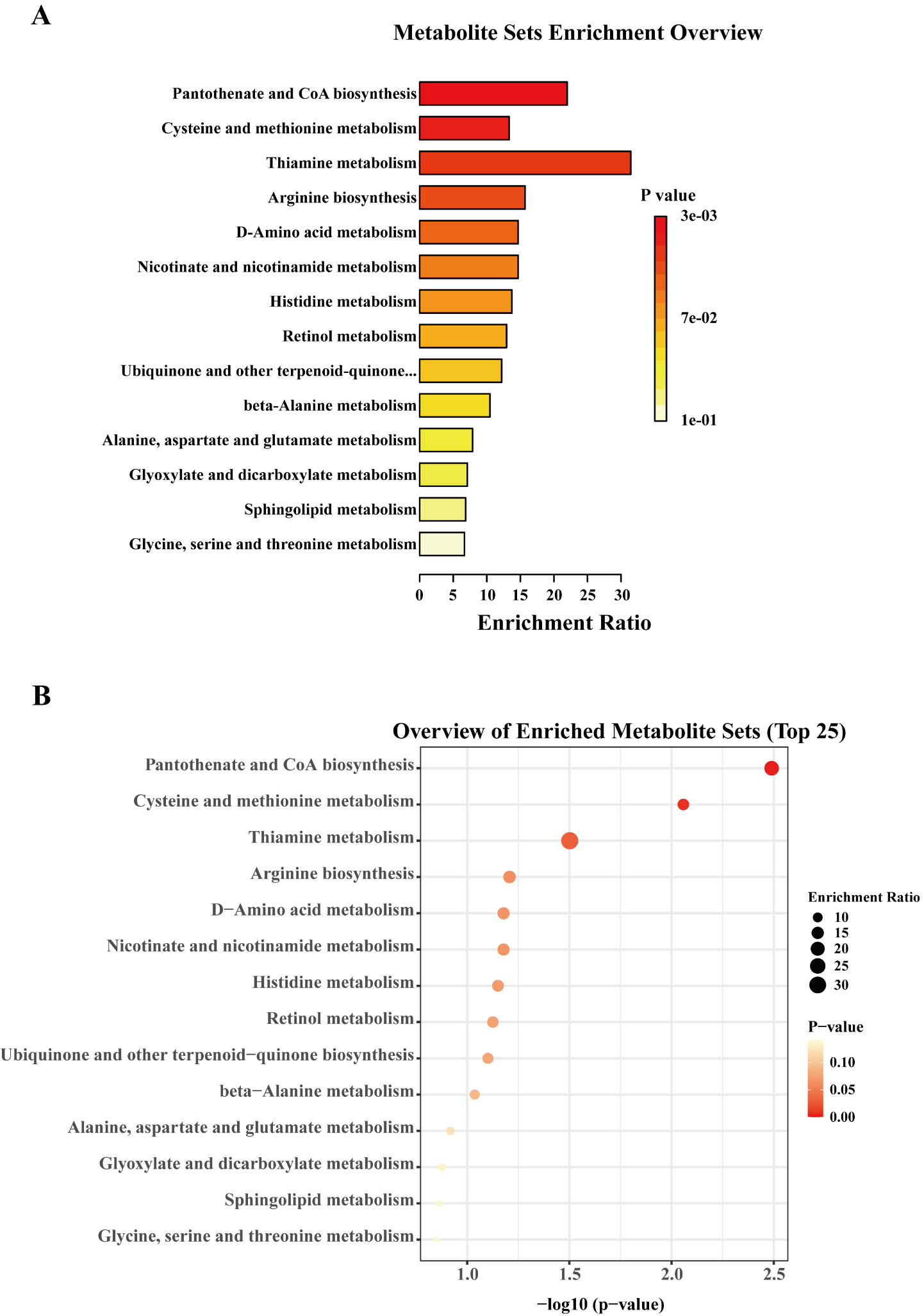


**Supplementary Figure 2B:** Overview of Enriched Metabolite Sets. (A) Metabolite Sets Enrichment Overview. This bar chart presents the enrichment ratios for the top enriched metabolite sets identified through pathway analysis. The y-axis lists the metabolite sets, while the x-axis shows the enrichment ratios. The color intensity of the bars indicates the level of enrichment, with darker red representing higher enrichment. The p-values on the right side highlight the statistical significance of the enrichment. The most significantly enriched pathways include pantothenate and CoA bio-synthesis, cysteine and methionine metabolism, and thiamine metabolism. (B) Overview of Enriched Metabolite Sets (Top 25). This scatter plot provides a detailed view of the top 25 enriched metabolite sets. The x-axis shows the statistical significance (-log10 p-value), while the y-axis lists the metabolite sets. The size and color of the circles indicate the enrichment ratio, with larger and darker circles representing higher enrichment. This visualization allows for a nuanced understanding of the relative importance and significance of the enriched pathways, with pantothenate, cysteine/methionine, and thiamine metabolism being the most prominently enriched.


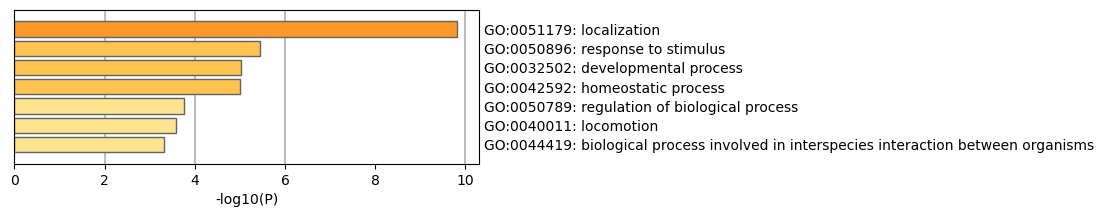


**Supplementary Figure 4A:** GO enrichment of L-cystine-associated genes. Hierarchical clustering of enriched parent GO terms (FDR < 0.05). Localization (L-cystine transport) and Homeostatic process (redox balance) exhibit highest significance (−log₁₀(P) > 6). Full terms: Localization (GO:0051179), Response to stimulus (GO:0050896), Developmental process (GO:0032502), Homeostatic process (GO:0042592), Regulation of biological process (GO:0050789), Locomotion (GO:0040011), Interspecies interaction (GO:0044419).


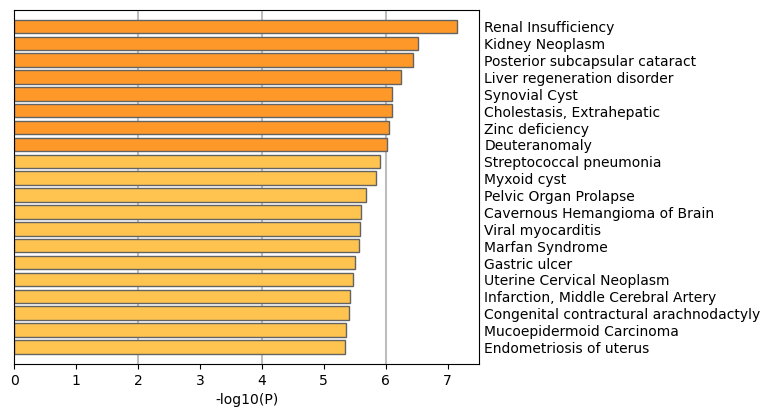


**Supplementary Figure 4B:** Disease association network of L-cystine-interacting genes. Heatmap showing significant disease terms (DisGeNET) enriched for L-cystine-associated genes. Top 20 terms ranked by -log₁₀(P-value) include: Renal Insufficiency, Kidney Neoplasm, Posterior subcapsular cataract, Liver regeneration disorder, and Cerebral Infarction. Color scale indicates enrichment significance. Diseases cluster into: Redox-failure disorders (cataract, viral myocarditis); Aging-associated degeneration (synovial cyst, pelvic prolapse);Maladaptation to damage (impaired liver regeneration, gastric ulcer).


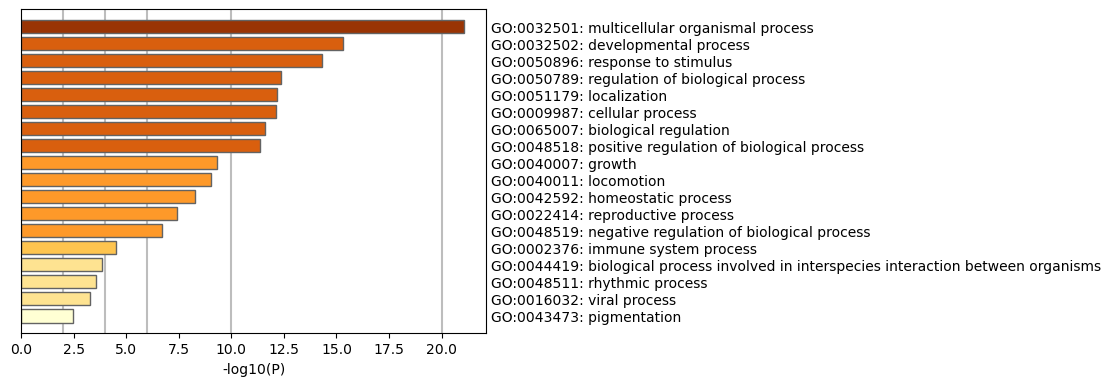


**Supplementary Figure 4C:** GO enrichment analysis of genes interacting with vitamin B5 (pantothenic acid). The bar chart depicts the –log10(P-value) for the most significantly enriched biological-process GO terms among the 18 terms with *P* < 0.05. Bars are arranged in descending order of statistical significance. Key processes include developmental regulation (GO:0032502, GO:0040007), response to external stimuli (GO:0050896), and homeostatic maintenance (GO:0042592), suggesting that vitamin B5 exerts pleiotropic effects on organismal growth, immunity, and metabolic balance.


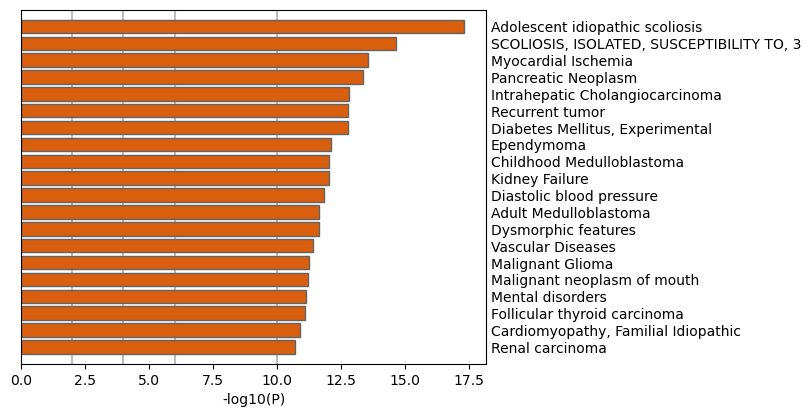


**Supplementary Figure 4D:** Disease association network of vitamin B5 (pantothenic acid)-interacting genes. Heatmap displaying the top 20 significantly enriched disease terms (DisGeNET) among genes reported to physically or genetically interact with vitamin B5. Terms are ranked by descending −log₁₀(P-value), with key associations including Adolescent idiopathic scoliosis, Myocardial ischemia, Pancreatic neoplasm, Intrahepatic cholangiocarcinoma, and Familial idiopathic cardiomyopathy. Color intensity reflects statistical significance. The enriched diseases stratify into three functional clusters: (i) Musculoskeletal and connective-tissue disorders (scoliosis, dysmorphic features); (ii) Onco-metabolic pathologies (pancreatic, renal, thyroid, and CNS malignancies); and (iii) Cardiorenal syndromes (myocardial ischemia, cardiomyopathy, kidney failure), supporting a pleiotropic role of vitamin B5 in regulating oxidative stress, energy metabolism, and tissue homeostasis.


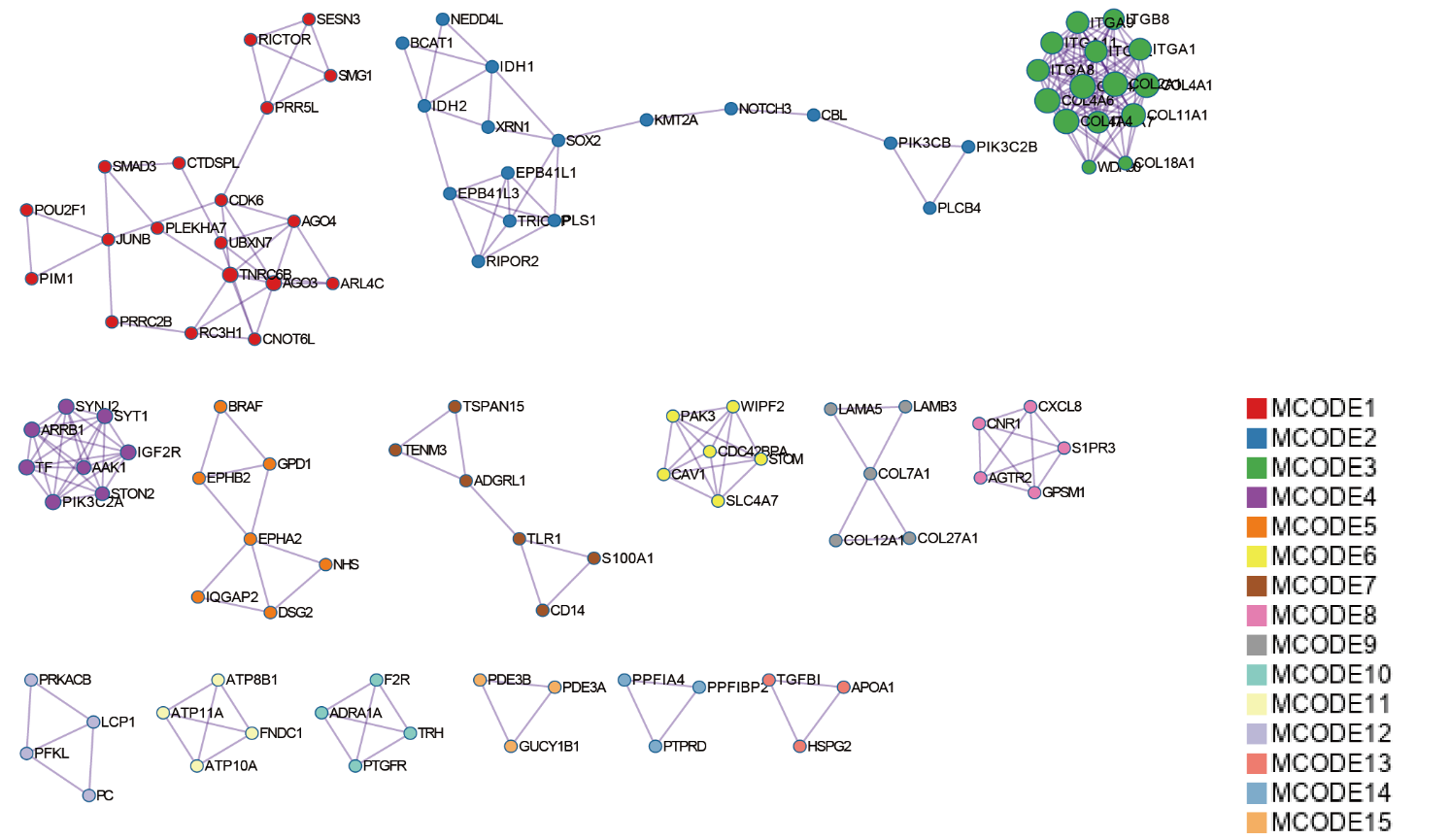


**Supplementary Figure 4E:** Vitamin B5-centric protein interactome reveals functional convergence on aging pathways. PPI network of Vitamin B5-associated genes colored by MCODE functional modules: MCODE 1: Oncogene-induced senescence (R-HSA-2559585) with TGF-β signaling (R-HSA-9839394); MCODE 3: Extracellular matrix (ECM) remodeling (hsa04512) and integrin signaling (R-HSA-216083); MCODE 5: Stress response regulation (GO:1903034) and ERK signaling (GO:0070372); MCODE 9: Collagen assembly (R-HSA-2022090) and matrisome organization (M5884). Node size reflects connectivity degree; edge thickness indicates STRING confidence score >0.7.


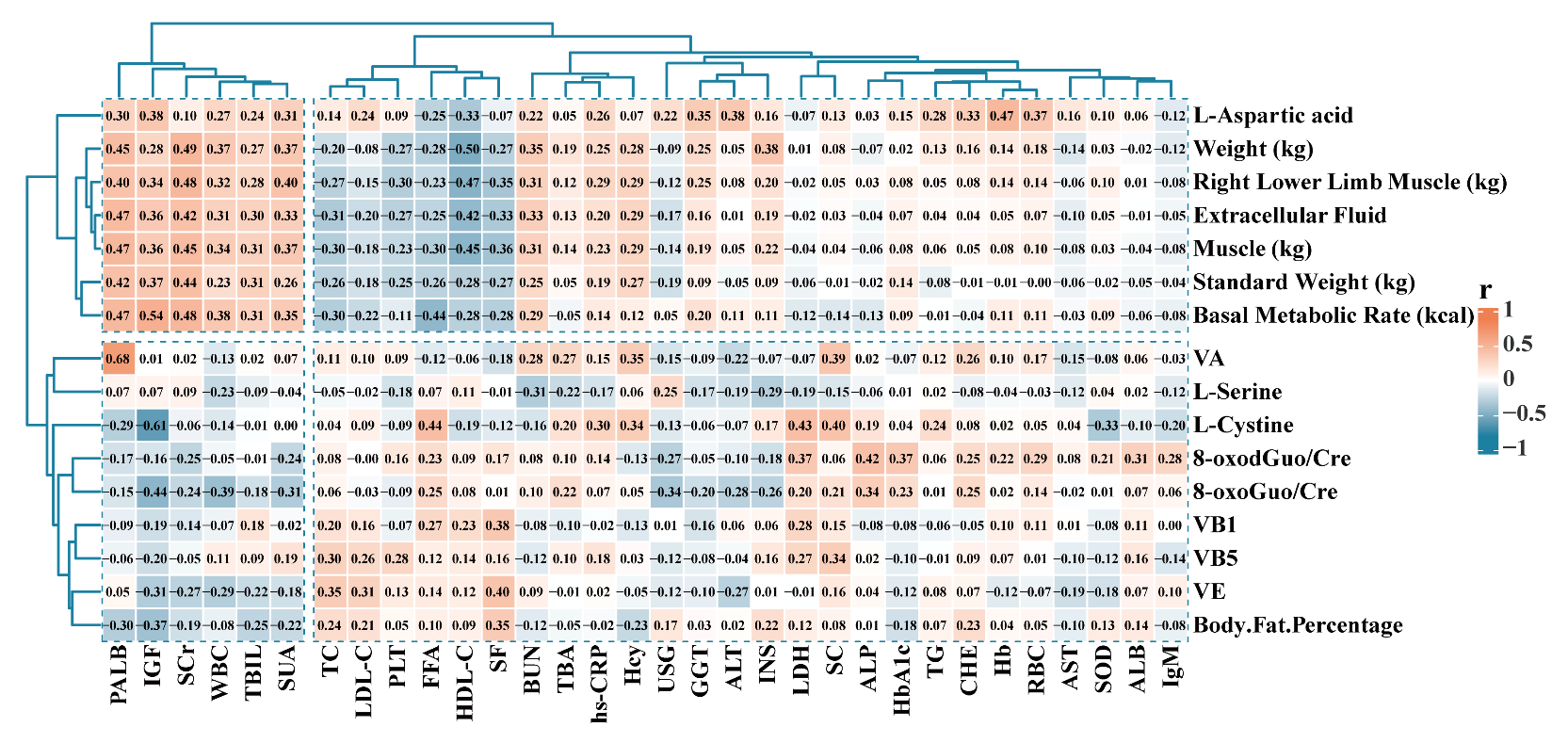


**Supplementary Figure 5C:** The heatmap shows how biochemical indicators relate to the full nutritional aging clock markers. The vertical axis represents full nutritional aging clock markers, while the horizontal axis represents biochemical indicators. A color gradient indicates the strength of the correlation coefficient, with blue representing a negative correlation and orange representing a positive correlation. The numbers in the squares represent the correlation coefficients.
